# Plant Cell Imaging Based on Nanodiamonds with Excitation-Dependent Fluorescence

**DOI:** 10.1186/s11671-016-1641-0

**Published:** 2016-09-23

**Authors:** Li-Xia Su, Qing Lou, Zhen Jiao, Chong-Xin Shan

**Affiliations:** School of Physics and Engineering, Zhengzhou University, No.75 Daxue Road, Zhengzhou, 450052 China

**Keywords:** Nanodiamond, Cell imaging, Fluorescence, Plant cell

## Abstract

Despite extensive work on fluorescence behavior stemming from color centers of diamond, reports on the excitation-dependent fluorescence of nanodiamonds (NDs) with a large-scale redshift from 400 to 620 nm under different excitation wavelengths are so far much fewer, especially in biological applications. The fluorescence can be attributed to the combined effects of the fraction of sp^2^-hybridized carbon atoms among the surface of the fine diamond nanoparticles and the defect energy trapping states on the surface of the diamond. The excitation-dependent fluorescent NDs have been applied in plant cell imaging for the first time. The results reported in this paper may provide a promising route to multiple-color bioimaging using NDs.

**Electronic supplementary material:**

The online version of this article (doi:10.1186/s11671-016-1641-0) contains supplementary material, which is available to authorized users.

## Background

Fluorescent nanodiamonds (NDs) have been investigated extensively for bioimaging, single-photon source, and drug delivery due to their remarkable optical properties, such as high photostability, facile surface functionalizability, aqueous dispersibility, good biocompatibility, and inertness [1–12]. To date, most of the bioimaging studies using fluorescent NDs are based on their color centers, especially nitrogen vacancy (N-V) centers [13–15]. However, the fluorescence efficiency of the color centers is usually low. Although some methods like ion implications and ion irradiating have been adopted to improve the fluorescence efficiency of color centers in NDs, cost-intensive techniques are always needed, which impede the applications of the fluorescent NDs in a large scale. In addition, the N-V centers in NDs are usually prepared by irradiation using a high-energy electron beam, proton beam, or helium ions, in which sophisticated equipment and complex experimental procedure are required [2, 16–18]. Therefore, finding a simple and cost-effective method for preparing fluorescent NDs is urgently needed. Recently, by taking advantage of the surface functionalization, researchers realized surface defect-related emission by modified NDs with various functional groups. For example, Mochalin et al. reported blue fluorescent NDs through wet chemistry route by conjugating carboxylated NDs with octadecylamine [19]. The fluorescence from functional groups can be excited by a common light source. However, the water solubility of such NDs is poor, which hinders the applications of the NDs in biotechnology. Xiao et al. have reported NDs with excitation-dependent fluorescence properties, and the fluorescence was attributed to the diverse functional groups residing on the NDs [20]. Nevertheless, the report on bioimaging using the excitation-dependent NDs has not been reported to date, which cast a shadow on whether such NDs can be employed in bioimaging. Moreover, theoretical calculations demonstrated that the fluorescence of carbon nanoparticles can also be adjusted by controlling the content of the sp^2^-hybridized carbons isolated by sp^3^ carbons and the size of the conjugated sp^2^ domains in isolated carbon nanoparticles [21, 22]. Nevertheless, experimental data are still absent currently. At present, most bioimaging research based on NDs is centered on animal cells, but the work associating plant cells and NDs is very rare. Therefore, it is meaningful to expand the application field based on NDs in plant word.

Herein, we reported an easy way to prepare NDs with excitation-dependent fluorescence, and the fluorescence can be attributed to the combined effects of the defect states on the surface of the NDs and sp^2^-hybridized domains. Synthetic type Ib diamond nanoparticles with a mean particle diameter of 50 nm were obtained by annealing at 420 °C for 30 min. The excitation-dependent fluorescent NDs have been applied in plant cell imaging for the first time. The results reported in this paper may provide a promising route for multiple-color bioimaging using NDs.

## Methods

### Synthesis of Fluorescent NDs

Synthetic type Ib diamond powders were purchased from Zhongnan Jete Superabrasives Co., Ltd (Zhengzhou, China). The NDs were sintered at 420 °C in air for 30 min to regulate the content of the sp^2^-hybridized carbon on the ND surface. The annealed NDs were dissolved in deionized water and separated by 8500 rpm centrifugation to remove the possible large agglomerates or impurities. The centrifuged NDs were then dispersed in deionized water (1 mg ml^−1^) for further research.

### Mung Bean Sprout Cultivation

Mung bean seeds having uniform size were placed in a Petri dish. Fifty milliliters of fluorescent NDs solution (1 mg ml^−1^) has been employed as the culture medium. The mung bean seeds were cultivated in the fluorescent NDs aqueous solution for 48 h at 25 °C in the dark until cotyledons emerged; after that, the spouts were rinsed by deionized water for five times to remove the possibly adsorbed NDs and contaminations. The control experiment was carried out in the same conditions except the culture medium was replaced by the deionized water.

### Imaging

The mung bean hypocotyl was sliced with freezing microtome and the thickness of the thin slice was about 40 mm. Confocal microscope images were taken to evaluate the cell imaging applications of the fluorescent NDs. Cell images were observed with a confocal laser scanning microscope (CLSM) Zesis 710 3-channels (Zesis, Germany) with the excitation wavelength at 405 nm, 488 nm and 480-550 nm, 600-680 nm of the emission filter.

### Characterization

The morphology and microstructure of the fluorescent NDs were characterized by a field emission scanning electron microscope (FESEM, JSM 6700F) and a high-resolution transmission electron microscope (HRTEM, FEI Technai G2 F20). The structural properties of the fluorescent NDs were characterized using a micro-Raman spectroscope (Renishaw RM 2000) and an X-ray diffractometer (XRD, PA National X’ Pert Pro). The Fourier transform infrared (FTIR) spectra of the NDs were recorded on a Bio-Rad Excalibur spectrometer (Bruker vector 22). A small amount of NDs was mixed with potassium bromide (KBr), grinded adequately, and then made into a pallet for a test. The absorption spectrum was measured by a Shimadzu UV-2401 instrument.

The fluorescence spectra of the NDs were measured by a double-grating spectrophotometer (Horiba FL-322). The transient fluorescence of the NDs was also measured by the Horiba FL-322 spectrometer using the following instrumental settings: 280 nm NanoLED; time range of 200 ns; peak preset at 5000 counts; repletion rate at 1 MHz; and synchronous delay of 50 ns.

## Results and Discussion

**Fig. 1:**
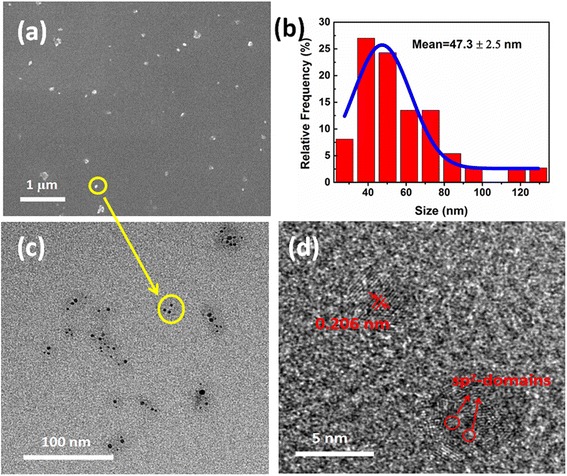
**a** *Top view* FESEM image of the NDs. **b** The particle size distributions of the NDs. **c** TEM images of the NDs. **d** Typical HRTEM images of the NDs

Figure 1a shows the SEM image of the NDs, and it is observed that the NDs have been dispersed well on the silicon substrate. The average size of the NDs was around 50 nm, as indicated in Fig. 1b. The HRTEM images show that these NDs are agglomerates of small-sized nanoparticles (Fig. 1c), which are composed of individual diamond nanocrystals with a size of 3–7 nm (Additional file 1: Figure S3). The lattice fringes of the nanocrystals can be clearly observed, as shown in Fig. 1d. The interlayer spacing is around 0.206 nm, which corresponds to the *d*-spacing of the diamond (111) planes. Another noteworthy phenomenon is that there are some shadows among the small-sized nanoparticles, as indicated in Fig. 1d, which may be sp^2^-hybridized carbons, and the size of conjugated sp^2^-domains was variational, which surrounds the sp^3^ carbon nucleus to form the agglomerates.

**Fig. 2:**
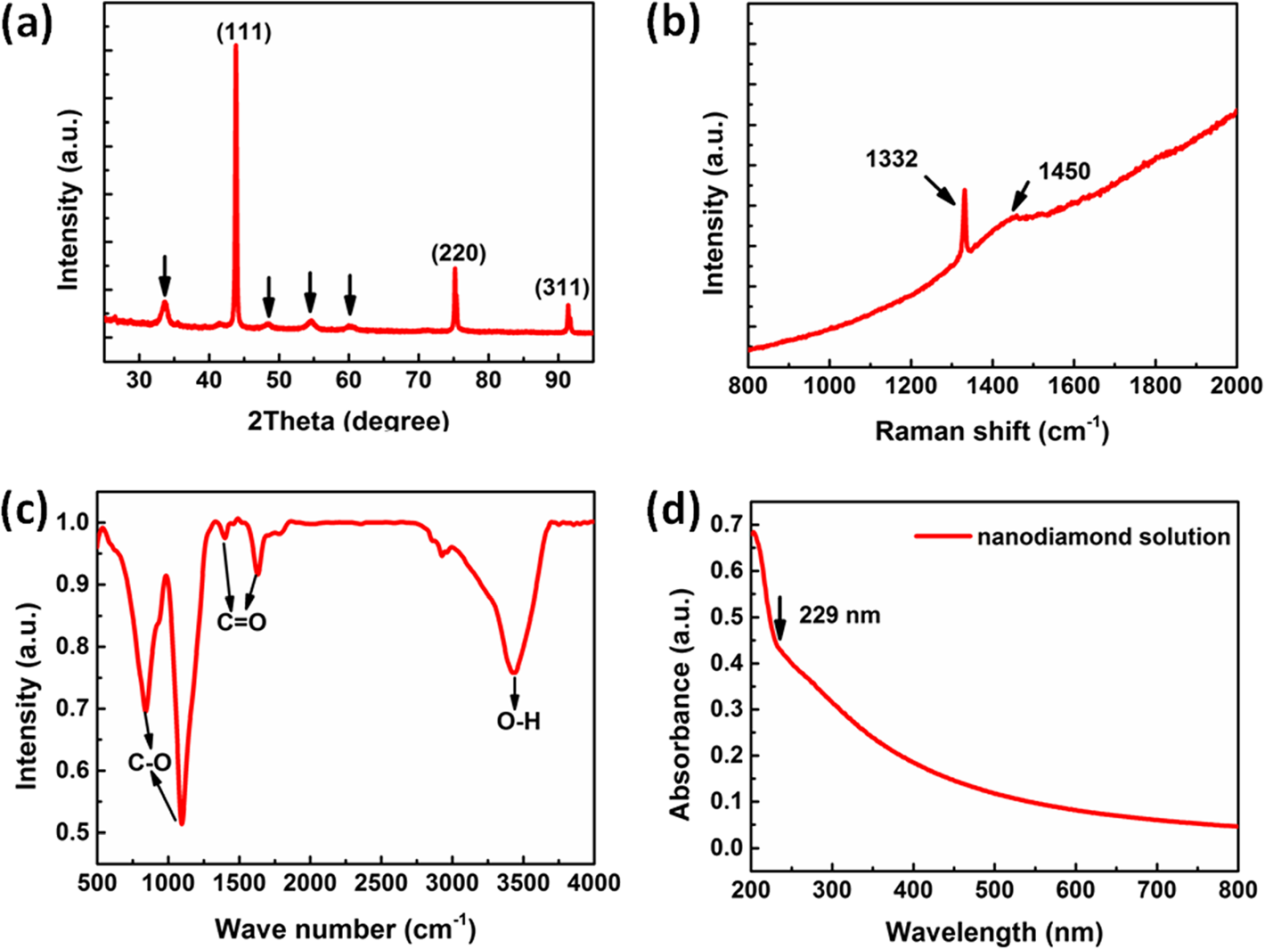
**a** The XRD spectrum of the NDs. **b** Raman spectra of the NDs. **c** FTIR spectra of the FNDs. **d** The absorption spectra of the NDs in solution

The XRD pattern of the NDs was shown in Fig. 2a, from which two sets of diffraction peaks can be observed. The distinctive peaks at around 43.8°, 75.2°, and 91.4° can be indexed to the diffraction from the (111), (220), and (311) planes of diamond, while the weak and broad peaks at around 33.6°, 48.4°, 54.4°, and 60.4° are corresponding to carbon-related materials. To further study the structure features of the NDs, the Raman spectrum of the NDs was measured at room temperature. As shown in Fig. 2b, two Raman peaks, locating at 1332 and 1450 cm^−1^, can be observed, respectively. The former can be attributed to the *D* band, while the latter to the *E* band of carbon. The *D* band is associated with T2G mode of sp^3^-bonded carbon, while the *E* band corresponds to transpolyacetylene segments at grain boundaries and surfaces, which can be attributed to sp^2^-hybridized carbon (with single hydrogen bonded to each carbon). Namely, the *E* band is caused by specific vibration modes (such as scissoring motion of the adamantane CH_2_ group) of adamantine [23]. A strong background can be seen in the spectra, which indicates that the NDs may have strong fluorescence.

To clarify the composition of the functional groups on the surface of the NDs, FTIR spectrum of the NDs has been measured, as shown in Fig. 2c. The vibration modes at around 800–1000 cm^−1^ can be attributed to the C–O stretching mode, while the peak at around 3450 cm^−1^ to the stretching vibration mode of the O–H bond [24]. The functional groups on the surface of the NDs may play an important role in determining the fluorescent properties of the NDs. The absorption spectrum of the NDs in aqueous solution is indicated in Fig. 2d. The spectrum shows a broad absorption extending to the visible region, and a well-defined peak appears at 229 nm (5.42 eV), which may be originated from the band edge absorption of diamond.

**Fig. 3:**
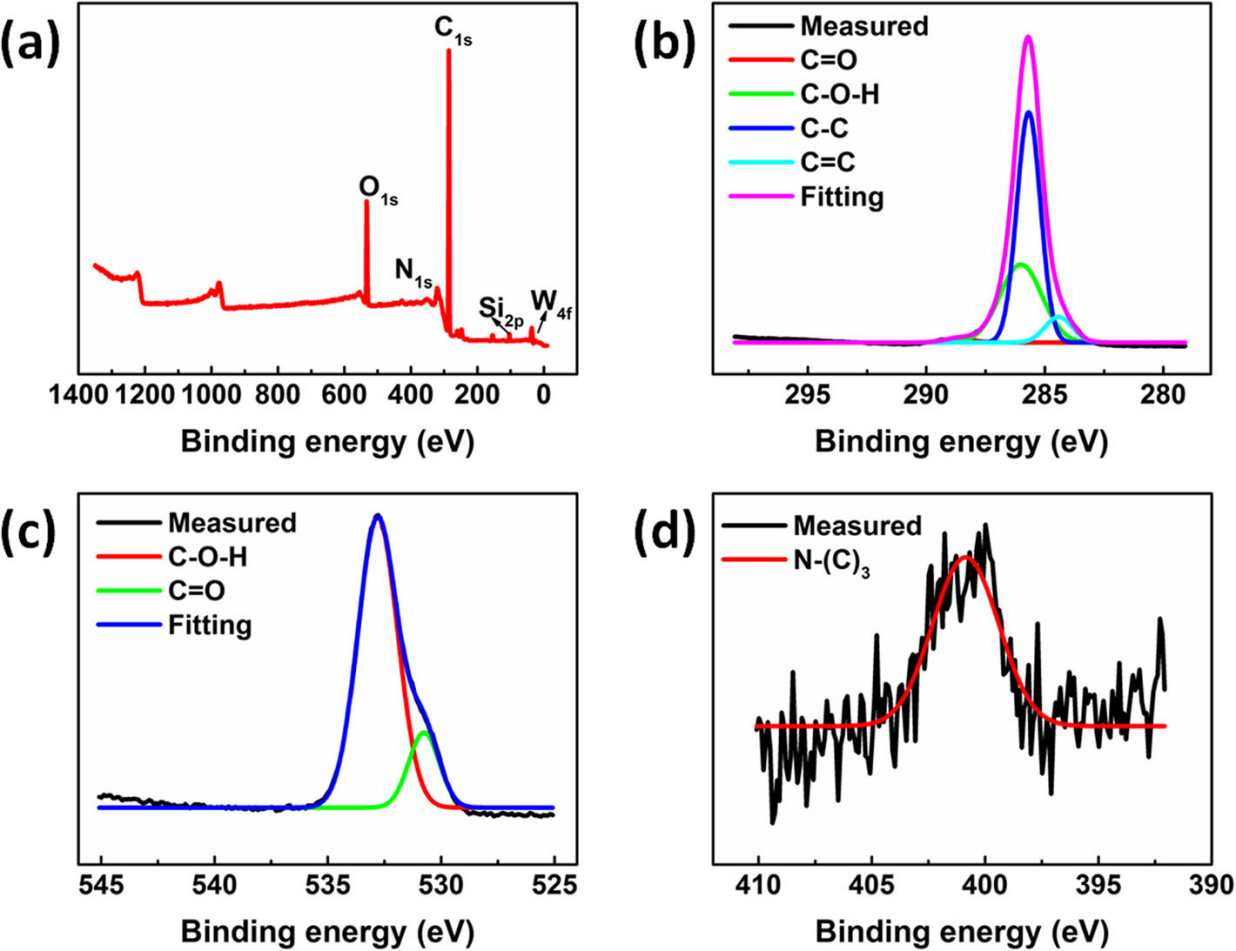
**a** XPS spectrum of the NDs, **b** C 1s spectra, **c** O 1s spectra, and **d** N 1s spectra of the NDs

As shown in the Fig. 3, the X-ray photoelectron spectroscopy (XPS) results display that the main carbon peak (C 1 s) at 285 eV, O 1 s peak at 532 eV, and N 1 s peak at 400 eV appeared in the full-survey XPS spectrum, consistent with the report by other researchers [25]. The element percentage of C 1 s, O 1 s, and N 1 s are estimated to be 85.67, 9.28, and 0.59 %, respectively. The impurity elements silicon (3.75 %) and tungsten (0.71 %) are found in the NDs. The XPS spectra with fine structures can be analyzed by decomposing the peak using the Gauss fitting method, as shown in Fig. 3b–d. The peak-fitting results shown in Fig. 3b reveal that the peak lain at around 284.6 eV is ascribed to graphitic sp^2^ C (C=C/C–C), the peaks centered at around 285.7 and 286.1 eV account for sp^3^ C (C–C, C–N) and sp^3^ C (C–O–H), and the peak at around 287.5 eV is due to the carbonyl C=O. The high-resolution O 1s XPS spectrum shown in Fig. 3c can be fitted into two Gaussian peaks at around 530.7 and 532.5 eV, which correspond to the carbonyl C=O and sp^3^ C (C–O–H) bonds, respectively. And, N 1s XPS peak at around 400.9 eV is attributed to the quaternary N (N–(C)_3_). Therefore, the results are further provided to verify the result of the FTIR that the functional groups like O–H bond can exist on the surface of the NDs after heat treatment.

**Fig. 4:**
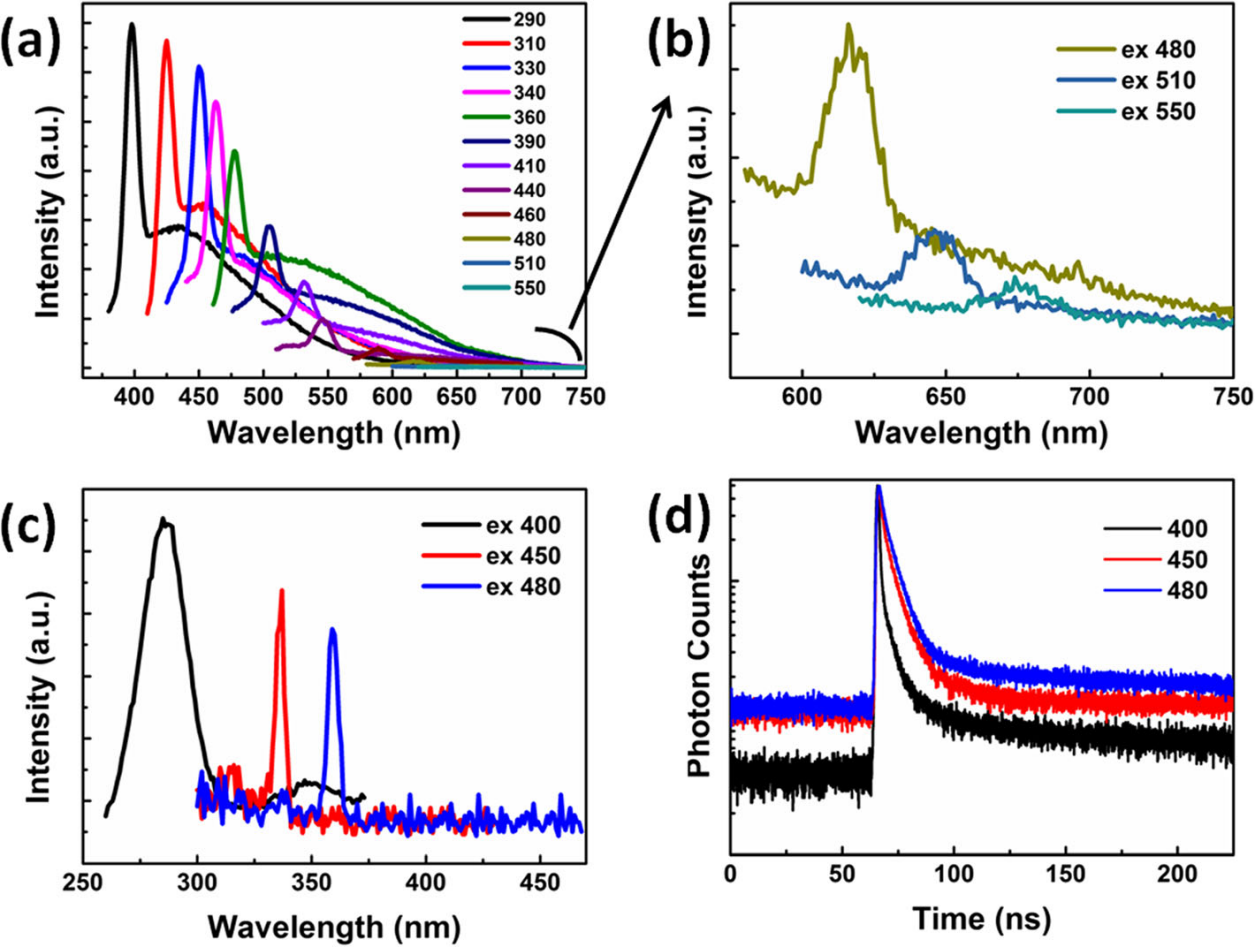
**a** Fluorescent emission spectra of NDs in solution. **b** Enlarged view of the fluorescence under the excitation of 480, 510, and 550 nm. **c** The excitation spectra of NDs in solution. **d** PL decay curve of NDs under 280-nm excitation detected at different wavelengths

Figure 4a presents the emission spectra of the NDs under different excitation emission wavelengths. And, the enlarged view of the fluorescence under the excitation of 480, 510, and 550 nm is shown in Fig. 4b. The fluorescence spectrum changes greatly when the excitation wavelength increases. Strong violet fluorescence was observed with a peak at 400 nm when the excitation wavelength is 290 nm. It should be noted that the emission peak redshifts from 400 to 620 nm, covering the visual color from blue to red. Similar phenomenon has been observed in carbon nanodots (CDs) [26, 27] and graphene quantum dots (GQDs) [22]. But, it is particularly worth mentioning that such a large-scale peak shift in the emission wavelength has been found for the first time compared with reported ND materials above [19–22]. Although excitation-dependent fluorescence is very typical for carbon nanomaterials, the origin of such phenomenon is still controversial. Xiao et al. have demonstrated that the fluorescence comes from the functional groups residing on the NDs, such as hydroxyl group, ketone C=O, and ester C=O groups. Tan et al. have revealed that the fluorescence of ultra-small NDs derives from highly localized π states or radiative recombination due to defect energy trapping states [28]. Mhasin et al. attributed the tunable fluorescence of GQDs to their size, edge configuration, shape, attached chemical functionalities, heteroatom doping, and defects [22]. In our case, the excitation-dependent fluorescence may be originated from the functional groups on the NDs. The fluorescent excitation spectra of NDs in solution were shown in Fig. 4c.

The transient fluorescence properties of the NDs are characterized with photoluminescence (PL) decay curves and shown in Fig. 4d, which were probed under the excitation wavelength at 280 nm and the different emission wavelengths at 400, 450, and 480 nm, respectively. The fluorescence decay kinetics for the NDs can be well fitted with a triple-exponential decay function. The decay fitting results are listed in Table 1. It can be found that the PL decay of the NDs were emission wavelength dependent, and radiative lifetime consists of three parts, *τ*_1_, *τ*_2_, and *τ*_3_ (where *A*_1_, *A*_2_, and *A*_3_ are the fractional contributions of the three fluorescence channels). The amplitude *A*_2_ with *τ*_2_ accounts for a large amount of the PL emission spectrum at short wavelength, and the amplitudes *A*_1_ and *A*_3_ with *τ*_1,_*τ*_3_ correspond to the emission peaked at long wavelength. The average lifetimes are calculated by *τ* = *A*_1_*τ*_1_ + *A*_2_*τ*_2_ + *A*_3_*τ*_3_, as 2.13, 5.03, 6.48 ns, respectively, which are different from those of N-V color centers (11.6 ns) [29]. The above data further indicates that the excitation-dependent fluorescence is originated from the surface states and sp^2^ domains but not the internal defects of the NDs. Therefore, we speculate that the observed excitation-dependent fluorescence of the NDs can be attributed to the synergetic effects of the fraction of sp^2^-hybridized carbon atoms and the defect energy trapping states on the surface of the NDs.

**Table 1:** Fitted decay times of the fluorescence of the NDs at different wavelengths under a 280-nm excitation

Ex/nm	Em/nm	*τ*_1_/ns	*A*_1_/%	*τ*_2_/ns	*A*_2_/%	*τ*_3_/ns	*A*_3_/%
280	400	1.0	32	1.0	55	9.7	13
	450	3.4	39	3.4	39.5	11.0	21.5
	480	4.1	34	5.3	49.1	14.7	16.9

Based on the above results, the possible mechanism for the excitation-dependent fluorescence from the NDs has been proposed. There exist various sizes of conjugated sp^2^ domains around the isolated sp^3^ ND cores. By increasing the sp^2^ bonding fraction and the size of the conjugated sp^2^ domains, the fluorescence induced by radiative recombination redshifts gradually. Meanwhile, the defect energy trapping states increase correspondingly, which may facilitate the occurrence of the nonradiative recombination. Therefore, the fluorescence peak of the NDs redshifts and the corresponding intensity diminishes gradually as the excitation wavelength increases.

**Fig. 5:**
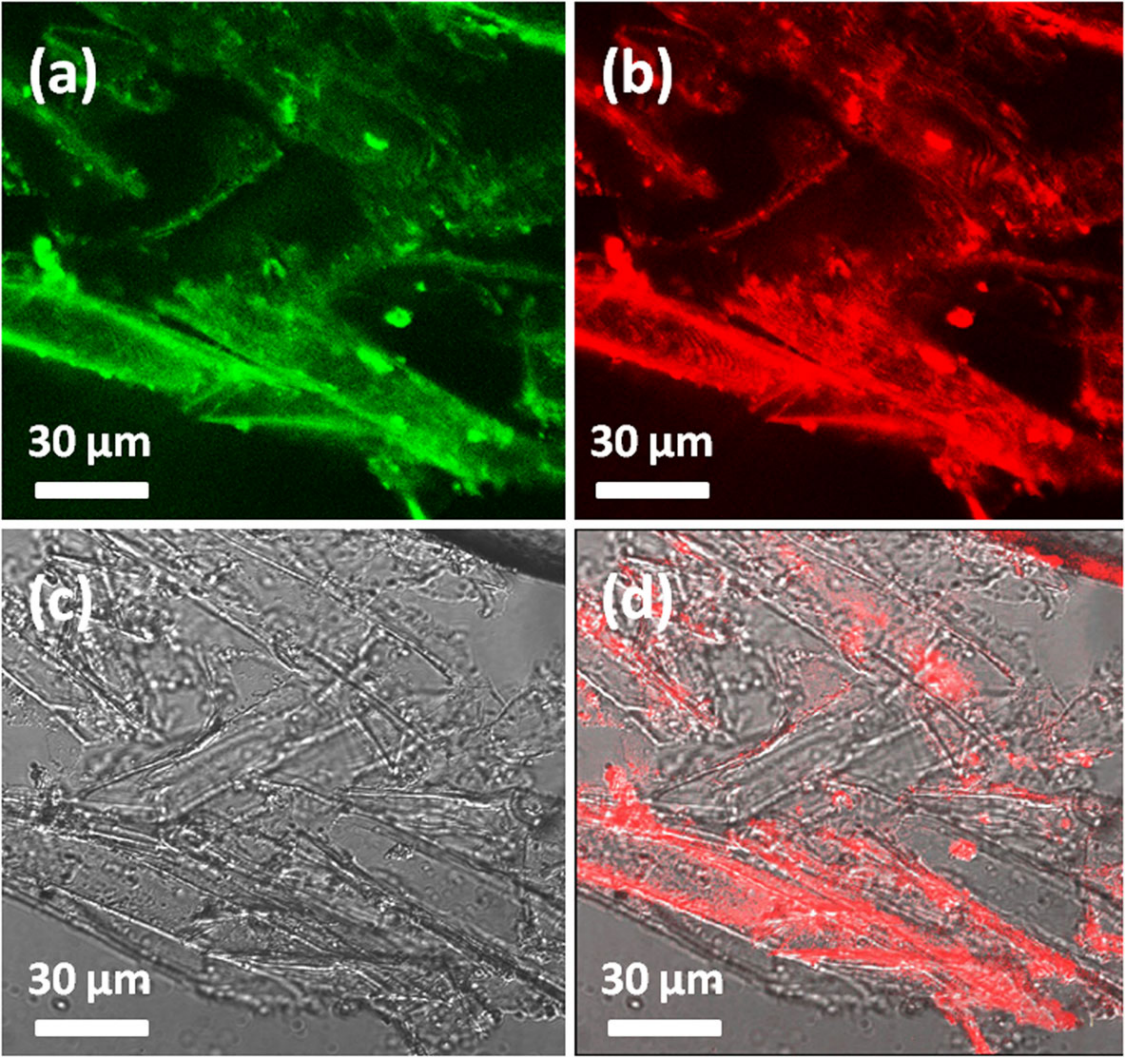
Confocal fluorescence images of mung bean stem cells cultivated in the solution with 1 mg mL^−1^ of NDs for 48 h. Panels **a**–**c** were obtained under excitations 405 nm, 488 nm, and bright field, respectively. **d** The overlayer of the images in **b** and **c** proved that NDs invaded the cell cytoplasm. The emissions were recorded in different ranges: **a** 480–550 nm and **b** 600–680 nm

In vitro cell imaging of the fluorescent NDs has been studied using confocal laser scanning microscopy (CLSM), as illustrated in Fig. 5, and the control experiment is shown in Additional file 1: Figure S6. There are only noise signals in the images of the control groups, which imply that the cells used in our experiment have no auto-fluorescence. The cell internalized fluorescent NDs can be excited by the 405- and 488-nm light from laser source, and green and red fluorescence can be emitted from the NDs. Obviously, the fluorescence areas are overlayed on the locations of the cells, as indicated from the Fig. 5d, which means that the fluorescent NDs could uptake by the cells and accumulated in the cells. From the CLSM images, bright points were also found within the cells, which maybe the cell nucleus. From the above data, we can draw the conclusion that the fluorescent NDs can be uptaken by the mung bean sprouts, and the NDs are mainly located at the cytoplasm and nucleus of the mung bean sprouts. This is the first report on the bioimaging of plant cells on the basis of this kind of excitation-dependent fluorescence from NDs.

## Conclusions

In conclusion, water dispersible fluorescent NDs have been prepared, and excitation-dependent fluorescence has been observed in the NDs. The excitation-dependent fluorescent NDs have been employed in mung bean cell imaging for the first time. Note that the excitation-dependent fluorescence may help to avoid the fluorescence interference of organism in plant and animal cells; thus, potential applications in high-quality cell imaging based on NDs may be achieved in the future.

## Additional File

Additional file 1: Supporting information: zeta potential, TEM, PL QY, and plantcell imaging characterization from nanodiamond samples. (DOCX 1983 kb)
