# Supplementary material for: Plant Cell Imaging Based on Nanodiamonds with Excitation-Dependent Fluorescence
Source: Nanoscale Res Lett. 2016 Sep 23;11:425. doi: 10.1186/s11671-016-1641-0 (PMC5035291; doi:10.1186/s11671-016-1641-0)
Supplement: Additional file 1: — Supporting information: zeta potential, TEM, PL QY, and plantcell imaging characterization from nanodiamond samples. (DOCX 1983 kb) [file 11671_2016_1641_MOESM1_ESM.docx]

Supporting Information

**Plant cell imaging based on nanodiamonds with excitation-dependent fluorescence**

Experimental detail:

Particle size distribution of 0.01 % wt. diamond nanoparticle colloidal in water have been measured using Zetasizer Nano ZS (Malvern Instruments).


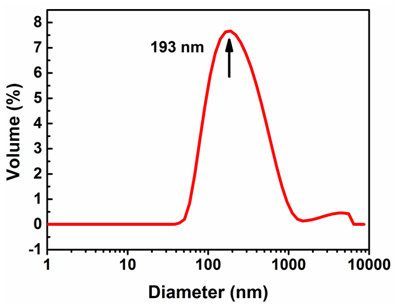


Figure S1. Particle size of NDs in water measured by Zetasizer Nano ZS.





Figure S2. The apparent zeta potential of the NDs in water measured by Zetasizer Nano ZS.


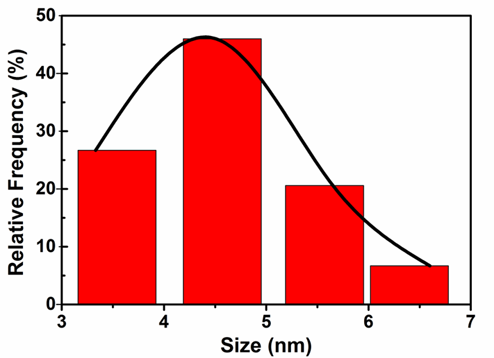


Figure S3. The particle size distribution of individual NDs measured using TEM.

The average size of NDs determined by DLS is about 193 nm, which is much larger that determined by TEM. DLS data includes the solvation shell, such as oxygen-containing groups, which makes the hydrodynamic size bigger. Besides, another possible reason for the results difference between TEM and DLS is aggregation.


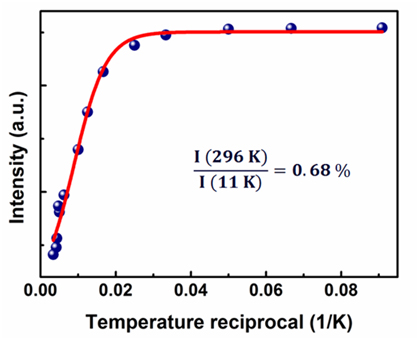


Figure S4. The spectrally integrated fluorescence intensity as a function of 1/*T*. At 296 K, *η*_int_ was 0.68%.


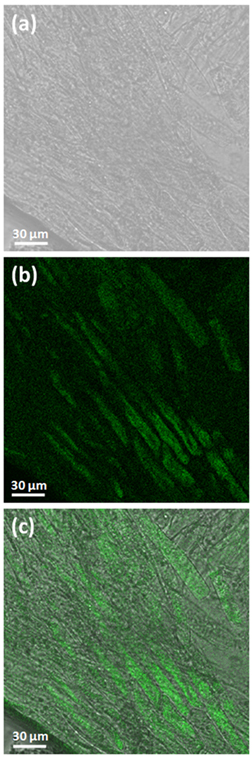


Figure S5. (a), (b) were confocal fluorescence images of mung bean stem cells cultivated with 1 mg mL^-1^ of NDs for 48 h under the bright field and 405 nm light excitation with the emissions recorded in rangs of 480-550 nm, respectively. (c) The overlayer of the images (a) and (b).

The morphology of the mung bean cells was columniform, as shown in Figure S5.


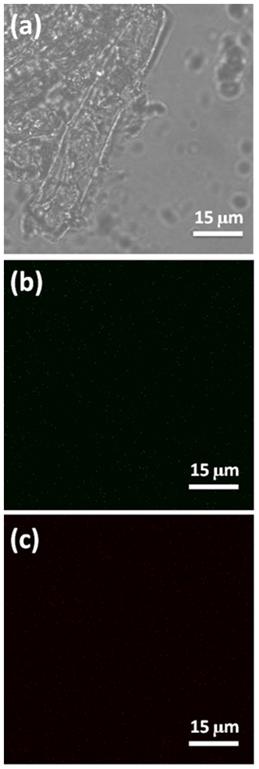


Figure S6. Confocal fluorescence images of mung bean stem cells treated with anything as normal control group. (a) The image was obtained under the bright field, (b), (c) were obtained under the excitations 405 nm and488 nm, respectively.

The images of the (b) and (c) were obtained under the 1000 volt of the photomultiplier tube, which was the highest working voltage. However, there were only noise signals in the images, which imply that the cells used in our experiment have no auto-fluorescence. The dimension of the single mung bean cell was about 70×10 micrometres.


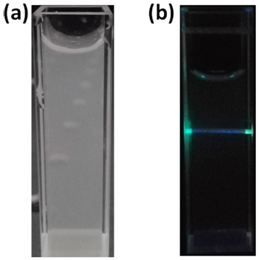


Figure S7. Photographs of NDs aqueous solution under (a) visible light, (b) 266 nm laser light excitation.

As shown in Figure S7, the NDs in water emit blue violet fluorescence excited by 266 nm laser.
